# Automatic processing of abstract musical tonality

**DOI:** 10.3389/fnhum.2014.00988

**Published:** 2014-12-09

**Authors:** Inyong Choi, Hari M. Bharadwaj, Scott Bressler, Psyche Loui, Kyogu Lee, Barbara G. Shinn-Cunningham

**Affiliations:** ^1^Center for Computational Neuroscience and Neural Technology, Boston UniversityBoston, MA, USA; ^2^Department of Biomedical Engineering, Boston UniversityBoston, MA, USA; ^3^Department of Psychology and Program in Neuroscience and Behavior, Wesleyan UniversityMiddletown, CT, USA; ^4^Graduate School of Convergence Science and Technology, Seoul National UniversitySuwon, South Korea

**Keywords:** music, tonality, markov-chain, ERP, P2

## Abstract

Music perception builds on expectancy in harmony, melody, and rhythm. Neural responses to the violations of such expectations are observed in event-related potentials (ERPs) measured using electroencephalography. Most previous ERP studies demonstrating sensitivity to musical violations used stimuli that were temporally regular and musically structured, with less-frequent deviant events that differed from a specific expectation in some feature such as pitch, harmony, or rhythm. Here, we asked whether expectancies about Western musical scale are strong enough to elicit ERP deviance components. Specifically, we explored whether pitches inconsistent with an established scale context elicit deviant components even though equally rare pitches that fit into the established context do not, and even when their timing is unpredictable. We used Markov chains to create temporally irregular pseudo-random sequences of notes chosen from one of two diatonic scales. The Markov pitch-transition probabilities resulted in sequences that favored notes within the scale, but that lacked clear melodic, harmonic, or rhythmic structure. At the random positions, the sequence contained probe tones that were either within the established scale or were out of key. Our subjects ignored the note sequences, watching a self-selected silent movie with subtitles. Compared to the in-key probes, the out-of-key probes elicited a significantly larger P2 ERP component. Results show that random note sequences establish expectations of the “first-order” statistical property of musical key, even in listeners not actively monitoring the sequences.

## Introduction

In music, properties such as pitch, harmony, and rhythm have statistically regular structures that build expectations about future events. Events such as notes or beats that violate such expectations are naturally salient and perceived as incongruent (Krumhansl, 1979; Schmuckler, 1989; Koelsch and Siebel, 2005; Huron and Margulis, 2010; Pearce et al., 2010). Many studies have shown that deviations of expectancy in various stimulus attributes elicit early event-related potential (ERP) components, known collectively as the mismatch negativity (MMN; Näätänen et al., 1978; Näätänen and Michie, 1979; Saarinen et al., 1992; see Näätänen et al., 2007 for review). Musical context can produce MMNs; an unexpected deviant single tone, chord, or even melodic pattern elicit ERP deviance components (Tervaniemi et al., 2003; Krohn et al., 2007) or an MMN (Alho et al., 1996; Tervaniemi et al., 1999). A few studies have demonstrated that violations of musical tonality can elicit MMNs. For instance, an out-of-key or an out-of-tune tone elicits a stronger early (~180 ms) frontal negative response compared to an in-key tone at the same position within an expected melody (Brattico et al., 2006). One study showed that occasionally replacing either the third or the last chord in a five-chord sequence with a musical chord that violates the established tonal context results in a negative ERP with a peak latency of 150–180 ms, topographically located in right anterior scalp sensors (Koelsch et al., 2000; Tillmann et al., 2003). This expectancy effect was stronger both when the irregular chord was in the final position in a sequence and when it was least expected given the musical context (Koelsch et al., 2000). The evoked ERP component was named the Early Right Anterior Negativity (ERAN), differentiated from the MMN by its timing and scalp distribution (Koelsch, 2009). Both the MMN and ERAN can be seen even if listeners are not actively attending to the ongoing note sequences (Brattico et al., 2006; Koelsch, 2009; Maidhof et al., 2010).

The P2 response, which is a positive peak at ~200 ms observed in front-central scalp electrodes is another example of a deviance-related evoked response. In visual priming paradigms, repeated object presentations suppress the P2 component (Wiggs and Martin, 1998; Gruber and Müller, 2005). Cuing also modulates the P2 response; the P2 component in response to a target visual object is enhanced if the target is preceded by an object from a different category (Freunberger et al., 2007). These studies suggest that the P2 component reflects neural processing associated with matching sensory inputs with stored memories (Freunberger et al., 2007). Consistent with this, an auditory cue-stimulus paradigm found that the auditory P2 is enhanced when the cue is invalid (i.e., when the stimulus violates cognitive expectation) (Golob et al., 2002). Auditory experiments show that the P2 response to non-target stimuli of an auditory oddball sequence is enhanced compared to responses to the same stimulus in the neutral condition (García-Larrea et al., 1992; Novak et al., 1992). Novak et al. interpreted the P2 enhancement as reflecting attention-modulated processing for the auditory discrimination task. More recently, Zhang et al. showed that P2 amplitude is suppressed in the responses to the later parts of repeated tone bursts (Zhang et al., 2009), which is interpreted within the context of predictive coding theory; the larger P2 reflects a mismatch between the sensory input and the predicted input based on past sensory events (Friston, 2005; Arnal and Giraud, 2012).

Statistical properties of the stimulus may define expectations that, when violated, generate ERP deviant responses. The statistical properties of the stimulus include zero-order probabilities, such as the event likelihood of particular notes, as well as first-order probabilities, such as the transitional probabilities from note to note. Sensitivity to these statistical properties are known to underlie musical expectation (Pearce et al., 2010; Loui, 2012); moreover, the transition probabilities between notes play an important role for tonality in Western music. Most past studies of musical expectation and violation used structured musical excerpts as stimuli; in such stimuli, probe tones had both low zeroth-order event frequencies and low transitional probabilities from other notes in the key (e.g., see Tillmann et al., 2003; Brattico et al., 2006; Peretz et al., 2009). As a result, it was not possible to separate the relative contributions of note frequency and “abstract” musical tonality (arising from integrating information across the sequence of notes over time) to the ERP deviant responses found in previous studies.

Moreover, in most musical priming studies, the timing of target events was fixed during repeated trials (Besson and Faïta, 1995). Temporal expectations (i.e., predictable onset times of target chords/notes) may also contribute to ERPs in response to perceptually unexpected events. Assuming that such temporal expectations interact with the encoding of sensory stimulation (Nobre et al., 2007; Costa-Faidella et al., 2011), and that predictive information processing (Rao and Ballard, 1999) plays a role in music cognition (Rohrmeier and Koelsch, 2012), it is likely that the temporal anticipation of probe notes influences, and potentially enhances, ERPs elicited by notes that are unexpected either because of their physical dissimilarity to or because of their abstract incongruity with the preceding context.

In this study, we focus on cortical responses to a musical event that violates its surrounding tonal context. Our aim was to isolate ERP effects elicited by manipulations of abstract melodic context by ensuring that other expectations (e.g., repeated melodic or temporal structure) are minimized. More specifically, our aims on the stimulus design were (1) to equalize the event frequencies of deviant notes to those of surrounding standard (in-key) notes; (2) to make the sequences temporally irregular; and (3) to make the temporal position of the deviant notes unpredictable. In order to achieve above aims, we used pseudo-random tone sequences generated by Markov chains. The use of Markov chains allowed us to manipulate note transition probabilities to establish the expectation of a musical key, while equating the event frequencies of probe notes that either were within or outside of the expected key. Sequences were from one of two different major diatonic scales separated by one-semitone (C major and C# major). The note transition probabilities were engineered to ensure a clear expectation of the major keys. In both of the keys (C major and C# major), notes corresponding to Western G# and F tones were used as the probe tones. Importantly, the F tone was an in-key note in both keys, but the G# was an out-of-key note in the C major context and an in-key note in the C# major context (that is, the tone sequences consisted of seven in-key tones and one out-of-key tone in the C major context, but seven in-key tones in the C# major context). To minimize the influence of temporal expectancy, we also made the tones in each sequence temporally irregular.

We compare ERP responses to the same G# tone presented in the two different keys, contrasting results with responses to the same F tone in the two keys. We hypothesize that abstract musical tonality is processed pre-attentively. Specifically, we expected responses to the F tone to be similar in the two contexts, while we expected responses to the G# note to elicit a larger (deviant) ERP response in the C major context (where it was outside of the established key) compared to the ERP responses to the same G# note in the C# major context (where it was part of the established key). To test the hypothesis that this kind of musical expectancy is processed automatically, we instructed subjects to ignore the auditory stimuli as they watched silent movies with subtitles. We did not have a specific hypothesis about exactly which ERP component might be elicited by the out-of-key deviant. Rather, we explored whether any ERP components that arise from early (earlier than 300 ms) cortical processing show differences in the responses to the probe notes in different scale contexts. Thus, rather than looking into a specific time window or a specific area of scalp, we recorded EEG signals over the whole scalp and performed sample-by-sample statistical analysis as a function of time to detect any differences in the ERP time courses for in-key vs. out-of-key notes.

We were also interested in how the responses to the incongruent tone change over the course of experiment. Previous studies show that some ERP components increase over time (Loui et al., 2009; Ross and Tremblay, 2009; Tremblay et al., 2009), whereas other studies show decreased responses to repeated stimuli (e.g., Altmann et al., 2008). We partitioned the data from each subject into the first and second halves of the experiment, and compared the resulting ERPs in the two halves of the sessions.

## Materials and methods

### Participants

Fourteen (eight male, aged 21–42, average 27.1) volunteers participated in the experiments. Prior to the main experiment, all subjects participated in hearing tests and were found to have normal hearing thresholds (dB HL less than 20 dB for all frequencies from 125–8000 Hz). None of the subjects reported any history of neurological disorder or disease. All subjects completed a musical experience survey. Some subjects (9 out of 14) had a history of musical-instrument training with Western tonal music (self-reported from survey, average 8.29 years), but none were professional musicians or music majors. All participants provided written informed consent approved by the Institutional Review Board (IRB) of Boston University; the approved IRB protocol allows us to perform EEG experiments to test auditory perception. Participants were compensated at the hourly rate of $25.

### Stimulus presentation

Thirteen tones in semitone relationships on the third octave of the Western tonal system (C3 through C4) were generated digitally using MATLAB (Mathworks Inc.). The fundamental frequency (f0) of the lowest (C3) tone was 261.63 Hz and the f0s of the higher tones were determined according to the equal-tempered scale (f0 = 261.63 Hz × 2^*n*/12^, *n* = 0, 2, …12). Each tone had six harmonics of equal amplitude, presented in cosine phase. The duration of each complex tone was 300 ms. Cosine-squared onset and offset ramps (5 ms duration) were applied to minimize spectral artifacts caused by temporal discontinuities.

**Figure 1:**
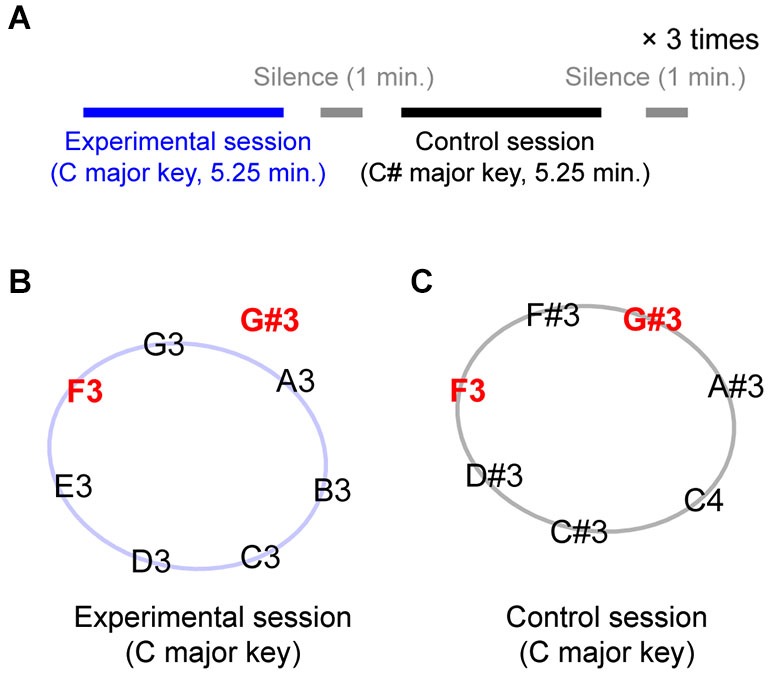
**Experimental design. (A)** A session structure. Experimental and control blocks were separated by one-minute silent break and alternately presented three times each. Each block went for 5.25 min. **(B)** Eight pitches presented during experimental blocks. Except G#, they all belonged to C major whole-tone diatonic scale. **(C)** Seven pitches presented during control blocks. They together made C# major scale.

The experiment consisted of 6 blocks: 3 experimental blocks and 3 control blocks, separated by one-minute silent breaks. The experimental and control blocks were alternately presented (see Figure 1A for the time structure); the experimental block was always presented first. Figures 1B,C show the tones that were presented during the experimental and control blocks, respectively. During each iteration of the experimental block, seven tones from a C major diatonic scale (C3-D3-E3-F3-G3-A3-B3, represented along the circle in Figure 1B) plus one out-of-key tone, G#3 (located outside the circle in Figure 1B), were repeatedly presented, making up a sequence of 350 notes. In the control block, the seven scale tones were selected from a C# major diatonic scale (C#3-D#3-F3-F#3-G#3-A#3-C4), up one semi-tone from the scale used in the experimental blocks. Note that in this condition, the G#3 tone, which was an out-of-key tone in the experimental block, was the melodically congruent dominant fifth of the C# major diatonic scale. Importantly, the F3 is an in-key tone for both experimental and control blocks (the subdominant fourth in the C major and mediant third in the C# major diatonic scale).

Tone sequences were generated from Markov chains that were constructed using the transition matrices shown in Tables 1, 2 for the experimental and the control blocks, respectively. The transition probabilities were carefully selected so that (1) the transition probabilities favored smaller intervals; (2) the tonic (first note in the scale) had a high probability of occurrence; and (3) minor-third transitions (e.g., from D to F, from E to G) were avoided to establish a major tonal context. To ensure that G#3 and F3 would occur with similar probabilities in both experimental and control blocks, we iteratively generated sequences of 350 tones until we found three pairs of experimental and control sequences that contained exactly the same number of G#3 and F3 tones. We used the same six sequences (three each of experimental and control) for all fourteen subjects. In total, G#3 occurred 143 times and F3 134 times out of 1,050 (3 × 350) tones in the experimental and in control blocks. Temporally adjacent tones were separated by a random silent interval equally distributed between 0.55 and 0.65 s in duration to make timing irregular and to reduce repetition artifacts in the EEG responses. Resulting event frequencies are shown in Table 3.

**Table 1:** **Transition probabilities for experimental block (C major key)**.

		Event k+1							
		C3	D3	E3	F3	G3	G#3	A3	B3
**Event k**	**C3**	0.2	0.2	0.1	0.2	0.2	0.1	0	0
	**D3**	0.2	0.2	0.2	0	0.1	0.1	0.1	0.1
	**E3**	0.2	0.2	0.2	0.1	0	0.1	0.1	0.1
	**F3**	0.2	0	0.1	0.2	0.2	0.1	0.1	0.1
	**G3**	0.3	0.1	0	0.1	0.2	0.1	0.1	0.1
	**G#3**	0.2	0.1	0.1	0.1	0.1	0.2	0.1	0.1
	**A3**	0.1	0.1	0.1	0.1	0.1	0.1	0.2	0.2
	**B3**	0.1	0.1	0.1	0.1	0.1	0.1	0.2	0.2

**Table 2:** **Transition probabilities for control block (C# major key)**.

		Event k+1						
		C#3	D#3	F3	F#3	G#3	A#3	C4
**Event k**	**C#3**	0.2	0.2	0.1	0.2	0.2	0.1	0
	**D#3**	0.3	0.2	0.2	0	0.1	0.1	0.1
	**F3**	0.2	0.2	0.2	0.2	0	0.1	0.1
	**F#3**	0.2	0	0.2	0.2	0.2	0.1	0.1
	**G#3**	0.3	0.1	0	0.1	0.2	0.2	0.1
	**A#3**	0.2	0.1	0.1	0.1	0.1	0.2	0.2
	**C4**	0.1	0.1	0.1	0.1	0.1	0.2	0.3

**Table 3:** **Resulting event frequencies**.

	C3	C#3	D3	D#3	E3	F3	F#3	G3	G#3	A3	A#3	B3	C4
C major	0.18	0	0.11	0	0.10	0.13	0	0.13	0.14	0.11	0	0.11	0
C# major	0	0.20	0	0.13	0	0.13	0.14	0	0.14	0	0.15	0	0.11

Sound stimuli were presented using Etymotic (Elk Grove Village, IL) ER-1 insert earphones connected to a Tucker-Davis Technologies (TDT, Alachua, FL) System 3 unit. The level of the stimulus presentation was 75 dB SPL. The TDT unit played back the sound stimuli (sampling frequency = 24414 Hz), and simultaneously provided timing signals for EEG recording. During stimulus presentation, subjects watched movies with the sound shut off and subtitles enabled (ignoring the auditory stimuli). All measures were obtained with the participants seated in an acoustically and electrically shielded booth (single-walled Eckel C-14 booth, Cambridge, MA).

### EEG acquisition and analysis

EEG data were collected using a Biosemi ActiveTwo system from 32 scalp electrode positions in the standard 10/20 configuration, using a sampling rate of 2048 Hz. Two additional reference electrodes were placed on the left and right mastoids. Timing signals sent from the TDT to mark stimulus events were recorded in an additional channel. The recordings were referenced to the average of the two mastoid electrode responses, then bandpass-filtered between 1–10 Hz using a 2048-point FIR filter applied offline. Matlab (Mathworks, Natick, MA) was used for EEG data processing and statistical analyses. EEGLab toolbox (Delorme and Makeig, 2004) was used for generating topographic plots.

For the G# and F tones, epochs from −100 ms to 700 ms relative to the onset of the tone were extracted for all electrode channels. The epochs from all channels were baseline-corrected using the mean value from −100 ms to 0 ms relative to the tone onset. The raw data were down-sampled to 256 Hz prior to analysis. After removing trials with EEG artifact (defined as voltages exceeding a threshold of ± 70 µV), epochs corresponding to each critical tone (G# or F) in each condition (experimental or control block) were averaged for each subject. The number of averaged epochs was constrained to be the same between conditions to ensure an evenly matched signal-to-noise ratio between conditions, and was determined by the condition with the fewest epochs remaining after artifact rejection. Across all subjects, this left between 73–110 epochs for F (mean was 91.0) and between 78–121 epoch for G# (mean 99.2).

## Results

### Comparing ERP time courses

**Figure 2:**
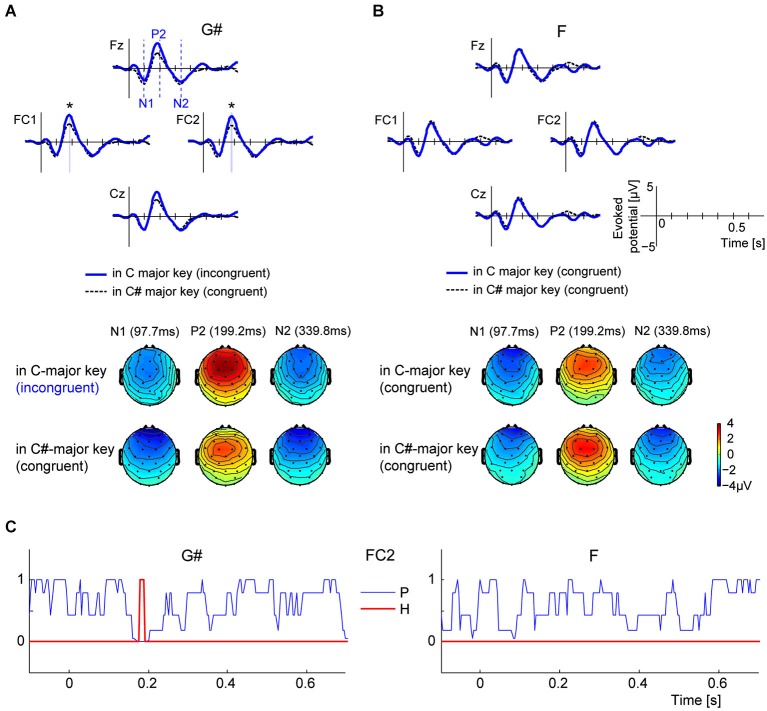
**Comparisons of ERP time courses in different contexts for G# (out of key in C major context, but in key in C# major context) and F (in key for both contexts). (A)** Comparing grand-average ERPs to the G# tone in C major context (where the G# is an out-of-key note) and C# major context (where the G# is an in-key note). Scalp topographies are computed at 97.7 ms, 199.2 ms, and 339.9 ms, respectively from left to right, correspondent to the timings of N1, P2, and N2 components (vertical dashed lines in the Fz axis indicate the three times). **(B)** Comparing grand-average ERPs to the F tone in C major context and C# major context. Note that F is an in-key note in both contexts. Scalp topographies show N1, P2, and N2 components at 97.7 ms, 199.2 ms, and 339.9 ms, respectively. **(C)** *P*-value time courses from Wilcoxon ranked-sign test for comparing instantaneous amplitudes of ERP pairs (blue lines). H (represented by red line) is 1 when *p* < 2.44 × 10^−4^ (= 0.05/205, 205 is number of time-samples under tests), and 0 otherwise. The period when *H* = 1 is also represented in **panel A**, denoted by blue shades and asterisks.

Figure 2A shows the grand-average ERP time courses elicited by G#, measured from four front-central electrodes (Fz, FC1, FC2, and Cz). The thick blue line shows ERPs elicited by the melodically incongruent G# in C major key (experimental blocks), while the thin dashed black line represents ERPs elicited by same G# tone from melodically congruent C# major key (control blocks). All four example ERPs have strong positive peaks around 200 ms (P2). Although the same physical stimulus evoked these ERPs in both key contexts, P2 amplitudes are larger when the stimulus is an incongruent, out-of-key tone than when it fits in the key context. Scalp topographies at three times (97.7 ms, 199.2 ms, and 339.8 ms) corresponding to the N1, P2, and N2 local-peak timings derived from electrode Cz are shown below the time-course ERPs. The topographies on the top row show scalp potentials elicited by the incongruent G# in the C major context, while the bottom topographies show potentials elicited by the congruous G# in the C# major context. At 199.2 ms, front-central electrodes show strong positive potentials, similar to the typical P2-pattern generated by equivalent current-dipole sources in the auditory cortices (Vaughan and Ritter, 1970). The front-central potential evoked by G# is stronger in the C major context than in the C# major context. In contrast, the ERPs elicited by F, an in-key note in both contexts, did not differ between conditions (see Figure 2B).

Statistical tests (Wilcoxon signed-rank tests) confirmed that the instantaneous amplitudes of ERPs to G# were larger when the note was incongruent with the key context than when it was congruent, but only around 190 ms after the tone onset (the time associated with the P2 response). These signed-rank tests were performed at each time sample from −100 ms to 700 ms (205 total time samples) using 14 individual-subject ERP pairs. The thin blue line in the left panel of Figure 2C shows the *p*-value time course computed using ERPs from channel FC2. The thick red line highlights the time course of a binary decision variable, H, which is 1 for p-values less than the Bonferroni-corrected α threshold (0.05/205 samples = 2.44 × 10^−4^), and 0 otherwise. Note that the null hypothesis can be rejected (*H* = 1) only between 180 and 190 ms for the G#. The decision variable H was determined by the above criterion of *p* < 2.44 × 10^−4^, which is a conservative criterion, equivalent to assuming that the instantaneous amplitude of ERP signal is independent at each time point (cf. Benjamini and Hochberg, 1995). In contrast, ERPs elicited by the F tone, which is melodically congruent in both the C major and C# major contexts, does not differ significantly in the two contexts at any time points regardless of electrode channel (see p and H time courses in the right panels of Figure 2C).

### Comparing ERP peak amplitudes

Peak amplitudes of the P2 component were measured by finding the local maximum within a fixed time window (150–230 ms) after note onset. Similarly, N1-peak amplitudes were computed by finding the local minimum within 80–120 ms after note onset. The P2 and N1 peak amplitudes were determined for each of the 32 electrode channels.

**Figure 3:**
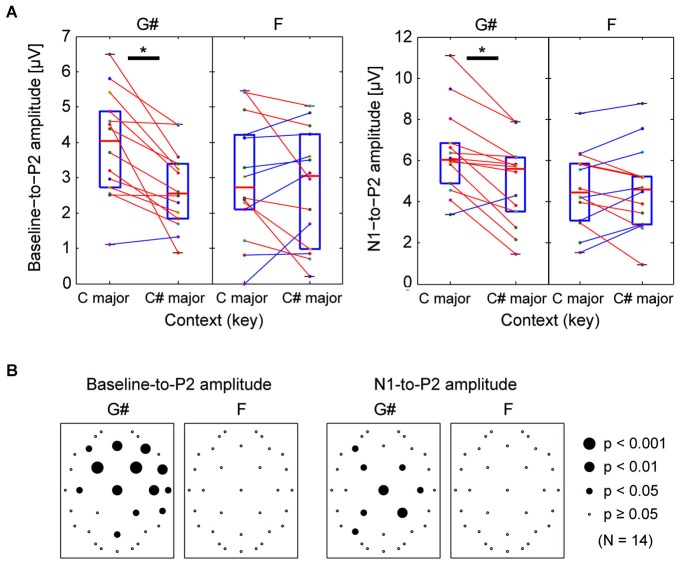
**Comparison of peak P2 ERP amplitudes for G# (out of key in C major context, but in key in C# major context) and F (in key for both contexts). (A)** Comparing individual listeners’ peak ERP amplitudes measured from Cz ERPs. Left panels show baseline-to-P2 peak amplitudes, while right panels show N1-to-P2 amplitudes. Each dot represents data from an individual subject. Lines connect individual subject results in the different contexts. Red lines are used for subjects whose amplitude was larger in the C major context, while blue lines are used for subjects whose amplitude was smaller in the C major context. Wilcoxon ranked-sign tests were performed; there was a statistically significant difference in the two contexts for the G#-elicited P2 and N1-to-P2 amplitudes; no significant difference was found from F-elicited ERP amplitudes. **(B)** The same statistical tests were performed for all 32 electrode locations. The resulting *p*-values are shown on scalp maps. Responses to G# were significantly affected by context in frontal-central sensor locations, but responses to *F* were not.

For channel Cz (where auditory evoked responses were large), each subject’s baseline-to-P2 amplitude is shown in Figure 3A. For the responses to G#, there was a significant difference between incongruent (C major) and congruent (C# major) contexts (Wilcoxon ranked-sign test, *p* = 0.0018), that is, the responses to G# was bigger in C major context; the Z-score for the enhancement effect was 1.12. However, for the F tone, changing contexts did not significantly influence the responses (same test, *p* = 1.00). The same is true if the P2 amplitude is quantified by calculating the N1-to-P2 amplitude; the N1-to-P2 amplitudes elicited by G# were significantly different depending on the surrounding note context (Wilcoxon ranked-sign test, *p* = 0.0018, *Z*-score = 0.82), but N1-to-P2 amplitudes elicited by F were not (*p* = 1.00).

Identical tests were performed for all other electrodes. Scalp distributions of these p-values are summarized in Figure 3B. Many of the front-central channels showed significant differences in the P2 responses to the G# tone in the two different tonal contexts. The scalp distribution of the effects is consistent with differences in neural activity at or near auditory cortex. In contrast, the F tone did not reveal many consistent differences in P2 responses between the two tonal contexts. When we used the N1-to-P2 peak amplitudes instead of baseline-to-P2 amplitudes, the *p*-values tended to be larger for the G#, and the differences shifted slightly towards central channels (see the right panels of Figure 3B).

### Comparing P2 amplitudes from the averages of the first and second-half epochs

**Figure 4:**
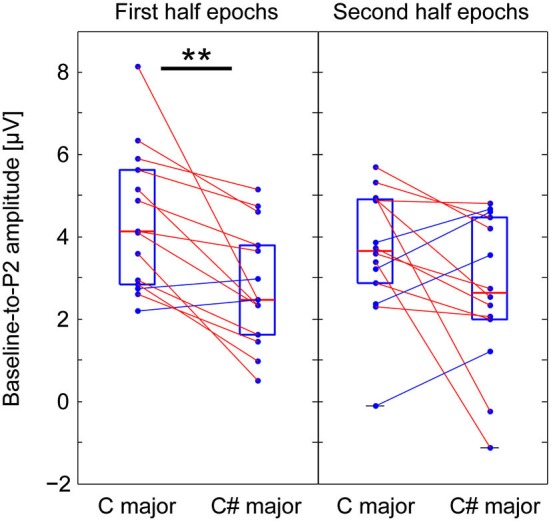
**Comparing P2 amplitudes from the averages of the first and second-half epochs to the G# (out of key in C major context, but in key in C# major context)**. Baseline-to-P2 amplitudes for Cz from the averages of the first and the second half epochs (left and right panels, respectively). A significant difference in the P2 amplitude between incongruent and congruent contexts was found from the first-half average (left), but not from the second-half average (right).

We were interested in how listener expectations are affected by context over time—specifically, we asked whether exposure to the Markov-chain stimuli might influence responses to contextual violations. To compare the effects of exposure on responses to context, we divided the epochs from each experimental and control session into two halves, and computed average ERPs separately across epochs from the first and the second halves of the sessions. The P2 peak amplitudes of responses to incongruent and congruent G# were then quantified as described above. For channel Cz, each subject’s baseline-to-P2 amplitude from the averages of the first and the second half epochs are shown in the left and right panels of Figure 4, respectively. A significant difference in the P2 amplitude between incongruent and congruent contexts was found from the first-half average (Wilcoxon—signed-rank test, *p* < 0.001), but not from the second-half average (the same test, *p* = 0.119).

### Influence of musical training on the P2 response to the incongruent tone

**Figure 5:**
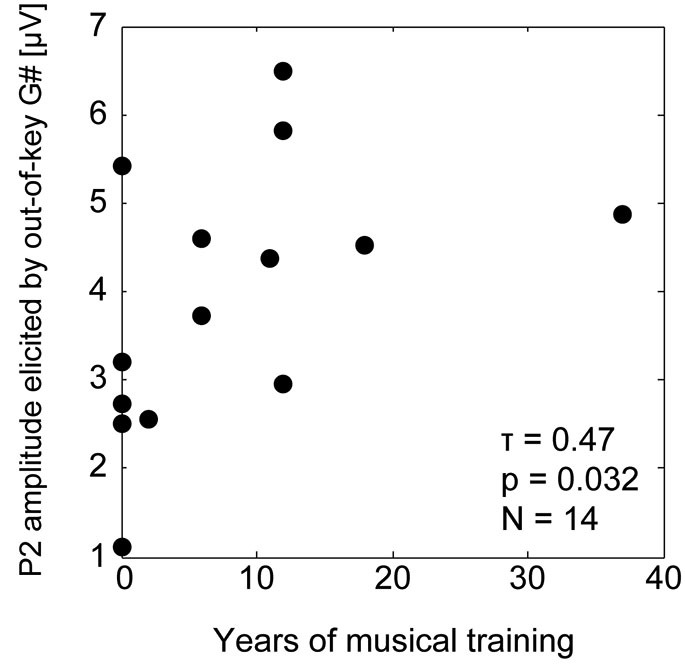
**Correlation between the musical training duration and the P2 amplitude for the response to out-of-key G#**.

We observed a relationship between the duration of individual subjects’ musical training and the P2 amplitudes of responses to the incongruent tones. The baseline-to-P2 amplitude (channel Cz) of the responses to out-of-key tones (G# in the C major context) was significantly positively correlated with self-reported years of musical training (rank correlation Kendall *τ* = 0.46, *p* = 0.032; see Figure 5). The musical training duration was not correlated with the P2 amplitudes for the responses to in-key tones (for G# in C# major context, *τ* = −0.034, *p* = 0.91; for F in C major, *τ* = 0.17, *p* = 0.44; for F in C# major, *τ* = −0.22, *p* = 0.32).

## Discussion

Here, we investigated human sensitivity to musical context in pitch. We compared EEG responses to probe tones (G# or F) in two different keys (C major and C# major) while subjects were watching a subtitled movie and ignoring tone sequences. To manipulate tonality statistically while controlling other musical properties (e.g., rhythm, temporal position of the target note, etc.), we used irregularly timed Markov chain-generated tone sequences in which pitch transitional probabilities were manipulated while event frequencies were controlled. The G# was an out-of-key tone in the C major context (established through the Markov chain) but an in-key tone in the C# major context. The F was an in-key tone in both keys. Results show that a front-central positive deflection peaking at ~200 ms is significantly greater for the out-of-key G# than the in-key G#. In contrast, responses to the F are not significantly different between the keys. Based on its scalp topographic patterns and latency, we identify the enhancement as part of the auditory P2 component.

Interpreting our results in the context of the past visual and auditory priming studies that also showed the P2 enhancement in the responses to incongruent stimuli (Wiggs and Martin, 1998; Golob et al., 2002; Gruber and Müller, 2005; Freunberger et al., 2007), we suggest that the repeated in-key tones in our experiment build up the tonal context of a particular key, so that an out-of-key tone is detected pre-attentively when it falls outside of the expected tonality. As described above, the previous semantic priming studies argue that the P2 component reflects the sensory-matching processing that compares the incoming stimulus with previously experienced inputs (Golob et al., 2002; Freunberger et al., 2007).

More insights into the P2 component can be obtained by considering its generator. Godey et al. used invasive EEG depth electrodes and scalp magnetoencephalographic scanning in the same subjects, and found that the P2 generators are localized to auditory association cortex including planum temporale (PT) and Brodmann Area 22 (Godey et al., 2001; for review, see Crowley and Colrain, 2004). PT has been known as a center for the early-stage cortical processing of language and complex sound such as music (Zatorre et al., 1998; Westbury et al., 1999; Marshall, 2000; Keenan et al., 2001; Wise et al., 2001). Griffiths and Warren argue that the PT is a “computational hub” that discriminates auditory objects and directs further processing in other cortical regions (Griffiths and Warren, 2002). This view is consistent with that of Novak et al., who claim that P2 enhancement reflects auditory processing during discrimination tasks and that the P2 induces later ERP components including P3, arising from other cortical regions (García-Larrea et al., 1992; Novak et al., 1992). Indeed, the P2 enhancement we find (Figure 2) has a shape similar to that of the ERP waveform reported by Zhang et al. (2009), which showed larger P2 amplitude in the response to the first tone in a sequence of repeated tone bursts. In the context of these reviews, we suggest that the P2 enhancement is a result of increased neural activity in PT elicited by an incongruent stimulus (e.g., G# in C major key) that violates some expected sensory property (“predictive coding theory,” Friston, 2005; Arnal and Giraud, 2012).

Previous studies show that P2 is sensitive to training (e.g., Ross and Tremblay, 2009; Tremblay et al., 2009). Recently studies showed that short-term musical training (Lappe et al., 2011), as well as long-term training (Seppänen et al., 2012), enhances P2. As exposure to the Markov-chain stimuli over the course of experiment is a form of training, one might expect the difference in the P2-amplitude across contexts to increase from the start of the experiment to its end (such as described in Loui et al. (2009)). However, our results show that, if anything, the P2 difference is more robust in the first half of an experimental session than the second. This finding suggests first that the P2 effect in this study builds up quickly; perhaps just a few notes are sufficient to set the musical key expectation, owing to the lifetime of exposure to Western diatonic scales tones that are ubiquitous in the culture. Additionally, the P2 might be sensitive to an adaptation effect to the exposure to the frequent G# tone (Zhang et al., 2009; Lanting et al., 2013), resulting in a reduction of the ERP amplitudes in conjunction with the diatonic-scale expectations.

Although we did not observe any short-term training effects on the P2 component, the amount of long-term musical training correlated with the ERP amplitude across individual subjects. Although the self-reported duration of musical training is a poor metric of musical ability, the results show that musical sophistication is related to the strength of tonality expectations observed here. Future studies could adopt more sophisticated ways of measuring musical ability, such as described in Müllensiefen et al. (2014), to better understand the relationship between musical ability and sensitivity to violations of tonality.

The main goal of this study, exploring ERP components that reflect automatic processing of musical tonality, is in line with the previous musical priming studies that showed negative-polarity ERP components (Koelsch et al., 2000; Tillmann et al., 2003; Brattico et al., 2006). However, the ERP patterns we find have polarity opposite that found in previous studies; there is little trace of any P2 enhancement in their results. Conversely, in our results, we found no enhancement of the strong negative deflection seen in their results, but rather an enhancement of the positive P2 deflection.

Differences in the stimulus and task designs could explain these differences. For example, Brattico et al. used multiple pre-defined melodies as stimuli, where a target in-key note was sometimes replaced with an out-of-key tone or an out-of-tune tone. Each folk melody they used was repeated four times over the course of their experiment; indeed, each melody was repeated even more often if one also counts the trials where the in-key tone of the same melody was replaced with an out-of-key or an out-of-tune tone. During the presentation of many in-key melodies, the out-of-key tone had lower event frequency than other notes. Koelsch et al., who reported ERAN as a response to musical violations, also used repeated stimuli with fixed musical structure where the target chords had smaller event frequencies (Koelsch et al., 2000). In contrast, in our study, melodies were generated from Markov chains that controlled for event frequencies of both in-key and out-of-key probe notes while manipulating transitional probabilities to establish clear musical key expectations. Thus, the out-of-key notes violated not a *specific* expectation for any particular note, but a *general* expectation of tonal context. By design, we ensured that only expectation violated by an out-of-key G# was the general property of tonality (in- vs. out-of-key). Another difference in our experimental design is that the melodies or chord sequences used in past studies were isochronous (temporally regular) so that the timing of the target tones could be predicted. As discussed above, temporal expectation has been shown to influence the encoding of sensory events (Nobre et al., 2007; Costa-Faidella et al., 2011). Both the repeatability of the specific melodies and the temporal expectation afforded by a regular rhythm could result in a strong MMN component (Näätänen et al., 2007). In contrast, in our experiment, the number of occurrences of the probe tones was the same in the out-of-key and in-key contexts, i.e., the effect of physical repetition (the zeroth-order statistic) was controlled. In addition, our stimuli did not have a fixed temporal structure; it was impossible to predict “when” the probe tone would appear. The only musical information delivered to the listeners was the tonality constructed by pitch transitions. We compared neural responses to physically identical tones presented in different tonal contexts, which suggests that the significant difference in the ERPs is solely due to differences in the tonal context and a high-level, abstract expectation for the notes likely to occur.

Despite the resulting ERP dissimilarities, it is inappropriate to conclude that the ERAN or MMN was not elicited in our paradigm, or that the P2 was not enhanced during Brattico’s or Koelsch’s experiments (Koelsch et al., 2000; Brattico et al., 2006). The observed scalp EEG is the sum of many ERP components; therefore, stronger components with opposite polarities may mask other components (Näätänen et al., 2011). Combining results from previous musical priming studies and semantic priming P2 studies (Wiggs and Martin, 1998; Golob et al., 2002; Gruber and Müller, 2005; Freunberger et al., 2007), it may be more appropriate to suggest that multiple neural mechanisms exist for detecting violations of tonality expectation, and that the relative contribution of each depends on the exact stimuli used to create the musical context and the expectancies that drive deviance detection.

Our experiment did not include behavioral ratings about the probe tones in the given diatonic scale contexts. Previous work shows that when listeners must make behavioral judgments, it affects the total ERP by eliciting components that pertain to decision-making and target responding, which may mask the ERPs related to perception and the sensitivity of tonal context (Koelsch and Siebel, 2005). Thus, in the present study we adopted a passive listening approach. This helps explain the absence of late ERPs such as P3a component or a late positive component in our EEG results, which have previously been shown to be evoked by an ending note that violates expectations created by the preceding melodic progression (Besson and Faïta, 1995; Patel et al., 1998). Nevertheless, behavioral ratings of incongruent pitch can be found from previous musical priming studies (e.g., Koelsch et al., 2000; Tillmann et al., 2003; Brattico et al., 2006). These studies show that pitches or chords that violate tonal context are salient, easily perceived, and rated as poor fitting. Future experiments with simultaneous behavioral and electrophysiological methods are planned to investigate late ERPs and subjects’ ability to detect out-of-key tones.

In summary, we investigated human sensitivity to tonal context during pitch processing by manipulating transitional probabilities while controlling for event frequencies in a Markov-chain generated, irregularly timed tonal context. Our main finding is that the auditory P2 indexes violation of tonal context, even in the absence of other regular musical structure. Our results differ from previous studies on musical priming; these differences may be attributable to temporal irregularity as well as the differentiation between zero-order and first-order statistical probabilities of the input. Viewed in the context of existing research, the present results suggest that multiple mechanisms, at the level of the auditory cortex and beyond, may be at work to give rise to the general sense of tonal expectation. Future work remains to be done to disentangle various aspects of the input that combine to give rise to musical expectations.

## Conflict of interest statement

The authors declare that the research was conducted in the absence of any commercial or financial relationships that could be construed as a potential conflict of interest.

## References

[B1] Alho K. Tervaniemi M. Huotilainen M. Lavikainen J. Tiitinen H. Ilmoniemi R. J.. (1996). Processing of complex sounds in the human auditory cortex as revealed by magnetic brain responses. Psychophysiology 33, 369–375. 10.1111/j.1469-8986.1996.tb01061.x 8753936

[B2] Altmann C. F. Nakata H. Noguchi Y. Inui K. Hoshiyama M. Kaneoke Y.. (2008). Temporal dynamics of adaptation to natural sounds in the human auditory cortex. Cereb. Cortex 18, 1350–1360. 10.1093/cercor/bhm166 17893422

[B3] Arnal L. H. Giraud A.-L. (2012). Cortical oscillations and sensory predictions. Trends Cogn. Sci. 16, 390–398. 10.1016/j.tics.2012.05.003 22682813

[B4] Benjamini Y. Hochberg Y. (1995). Controlling the false discovery rate: a practical and powerful approach to multiple testing. J. R. Stat. Soc. Ser. B 57, 289–300.

[B5] Besson M. Faïta F. (1995). An event-related potential (ERP) study of musical expectancy: comparison of musicians with nonmusicians. J. Exp. Psychol. Hum. Percept. Perform. 21, 1278–1296 10.1037//0096-1523.21.6.1278

[B6] Brattico E. Tervaniemi M. Näätänen R. Peretz I. (2006). Musical scale properties are automatically processed in the human auditory cortex. Brain Res. 1117, 162–174. 10.1016/j.brainres.2006.08.023 16963000

[B7] Costa-Faidella J. Baldeweg T. Grimm S. Escera C. (2011). Interactions between “what” and “when” in the auditory system: temporal predictability enhances repetition suppression. J. Neurosci. 31, 18590–18597. 10.1523/JNEUROSCI.2599-11.2011 22171057 PMC6623902

[B8] Crowley K. E. Colrain I. M. (2004). A review of the evidence for P2 being an independent component process: age, sleep and modality. Clin. Neurophysiol. 115, 732–744. 10.1016/j.clinph.2003.11.021 15003751

[B9] Delorme A. Makeig S. (2004). EEGLAB: an open source toolbox for analysis of single-trial EEG dynamics including independent component analysis. J. Neurosci. Methods 134, 9–21. 10.1016/j.jneumeth.2003.10.009 15102499

[B10] Freunberger R. Klimesch W. Doppelmayr M. Höller Y. (2007). Visual P2 component is related to theta phase-locking. Neurosci. Lett. 426, 181–186. 10.1016/j.neulet.2007.08.062 17904744

[B11] Friston K. (2005). A theory of cortical responses. Philos. Trans. R. Soc. Lond. B Biol. Sci. 360, 815–836. 10.1098/rstb.2005.1622 15937014 PMC1569488

[B12] García-Larrea L. Lukaszewicz A. Mauguiére F. (1992). Revisiting the oddball paradigm. Non-target vs neutral stimuli and the evaluation of ERP attentional effects. Neuropsychologia 30, 723–741. 10.1016/0028-3932(92)90042-k 1407488

[B13] Godey B. Schwartz D. de Graaf J. B. Chauvel P. Liégeois-Chauvel C. (2001). Neuromagnetic source localization of auditory evoked fields and intracerebral evoked potentials: a comparison of data in the same patients. Clin. Neurophysiol. 112, 1850–1859. 10.1016/s1388-2457(01)00636-8 11595143

[B14] Golob E. J. Pratt H. Starr A. (2002). Preparatory slow potentials and event-related potentials in an auditory cued attention task. Clin. Neurophysiol. 113, 1544–1557. 10.1016/s1388-2457(02)00220-1 12350430

[B15] Griffiths T. Warren J. (2002). The planum temporale as a computational hub. Trends Neurosci. 25, 348–353. 10.1016/s0166-2236(02)02191-4 12079762

[B16] Gruber T. Müller M. M. (2005). Oscillatory brain activity dissociates between associative stimulus content in a repetition priming task in the human EEG. Cereb. Cortex 15, 109–116. 10.1093/cercor/bhh113 15238442

[B17] Huron D. Margulis E. H. (2010). “Musical expectancy and thrills,” in Handbook of Music and Emotion: Theory, Research, Applications, eds Juslin P. N. Sloboda J. A. (New York, NY: Oxford University Press), 575–604.

[B18] Keenan J. P. Thangaraj V. Halpern A. R. Schlaug G. (2001). Absolute pitch and planum temporale. Neuroimage 14, 1402–1408. 10.1006/nimg.2001.0925 11707095

[B19] Koelsch S. (2009). Music-syntactic processing and auditory memory: similarities and differences between ERAN and MMN. Psychophysiology 46, 179–190. 10.1111/j.1469-8986.2008.00752.X 19055508

[B20] Koelsch S. Gunter T. Friederici A. D. Schröger E. (2000). Brain indices of music processing: “nonmusicians” are musical. J. Cogn. Neurosci. 12, 520–541. 10.1162/089892900562183 10931776

[B21] Koelsch S. Siebel W. A. (2005). Towards a neural basis of music perception. Trends Cogn. Sci. 9, 578–584. 10.1016/j.tics.2005.10.001 16271503

[B22] Krohn K. I. Brattico E. Välimäki V. Tervaniemi M. (2007). Neural representations of the hierarchical scale pitch structure. Music Percept. 24, 281–296 10.1525/mp.2007.24.3.281

[B23] Krumhansl C. L. (1979). The psychological representation of musical pitch in a tonal context. Cogn. Psychol. 11, 346–374 10.1016/0010-0285(79)90016-1

[B24] Lanting C. P. Briley P. M. Sumner C. J. Krumbholz K. (2013). Mechanisms of adaptation in human auditory cortex. J. Neurophysiol. 110, 973–983. 10.1152/jn.00547.2012 23719212 PMC3742970

[B25] Lappe C. Trainor L. J. Herholz S. C. Pantev C. (2011). Cortical plasticity induced by short-term multimodal musical rhythm training. PLoS One 6:e21493. 10.1371/journal.pone.0021493 21747907 PMC3126826

[B26] Loui P. (2012). “Statistical learning—what can music tell us?,” in Statistical Learning and Language Acquisition, eds Rebuschat P. Williams J. (Boston/Berlin: Walter de Gruyter), 433–462.

[B27] Loui P. Wu E. H. Wessel D. L. Knight R. T. (2009). A generalized mechanism for perception of pitch patterns. J. Neurosci. 29, 454–459. 10.1523/JNEUROSCI.4503-08.2009 19144845 PMC2779050

[B28] Maidhof C. Vavatzanidis N. Prinz W. Rieger M. Koelsch S. (2010). Processing expectancy violations during music performance and perception: an ERP study. J. Cogn. Neurosci. 22, 2401–2413. 10.1162/jocn.2009.21332 19702473

[B29] Marshall J. C. (2000). Planum of the apes: a case study. Brain Lang. 71, 145–148. 10.1006/brln.1999.2236 10716831

[B30] Müllensiefen D. Gingras B. Musil J. Stewart L. (2014). The musicality of non-musicians: an index for assessing musical sophistication in the general population. PLoS One 9:e89642. 10.1371/journal.pone.0089642 24586929 PMC3935919

[B31] Näätänen R. Gaillard A. Mäntysalo S. (1978). Early selective-attention effect on evoked potential reinterpreted. Acta Psychol. (Amst) 42, 313–329. 10.1016/0001-6918(78)90006-9 685709

[B32] Näätänen R. Kujala T. Winkler I. (2011). Auditory processing that leads to conscious perception: a unique window to central auditory processing opened by the mismatch negativity and related responses. Psychophysiology 48, 4–22. 10.1111/j.1469-8986.2010.01114.x 20880261

[B33] Näätänen R. Michie P. T. (1979). Early selective-attention effects on the evoked potential: a critical review and reinterpretation. Biol. Psychol. 8, 81–136. 10.1016/0301-0511(79)90053-x 465623

[B34] Näätänen R. Paavilainen P. Rinne T. Alho K. (2007). The mismatch negativity (MMN) in basic research of central auditory processing: a review. Clin. Neurophysiol. 118, 2544–2590. 10.1016/j.clinph.2007.04.026 17931964

[B35] Nobre A. Correa A. Coull J. (2007). The hazards of time. Curr. Opin. Neurobiol. 17, 465–470. 10.1016/j.conb.2007.07.006 17709239

[B36] Novak G. Ritter W. Vaughan H. G. (1992). Mismatch detection and the latency of temporal judgments. Psychophysiology 29, 398–411. 10.1111/j.1469-8986.1992.tb01713.x 1410172

[B37] Patel A. D. Gibson E. Ratner J. Besson M. Holcomb P. J. (1998). Processing syntactic relations in language and music: an event-related potential study. J. Cogn. Neurosci. 10, 717–733. 10.1162/089892998563121 9831740

[B38] Pearce M. T. Ruiz M. H. Kapasi S. Wiggins G. A. Bhattacharya J. (2010). Unsupervised statistical learning underpins computational, behavioural and neural manifestations of musical expectation. Neuroimage 50, 302–313. 10.1016/j.neuroimage.2009.12.019 20005297

[B39] Peretz I. Brattico E. Järvenpää M. Tervaniemi M. (2009). The amusic brain: in tune, out of key and unaware. Brain 132, 1277–1286. 10.1093/brain/awp055 19336462

[B40] Rao R. P. Ballard D. H. (1999). Predictive coding in the visual cortex: a functional interpretation of some extra-classical receptive-field effects. Nat. Neurosci. 2, 79–87. 10.1038/4580 10195184

[B41] Rohrmeier M. A. Koelsch S. (2012). Predictive information processing in music cognition. A critical review. Int. J. Psychophysiol. 83, 164–175. 10.1016/j.ijpsycho.2011.12.010 22245599

[B42] Ross B. Tremblay K. (2009). Stimulus experience modifies auditory neuromagnetic responses in young and older listeners. Hear. Res. 248, 48–59. 10.1016/j.heares.2008.11.012 19110047 PMC2668103

[B43] Saarinen J. Paavilainen P. Schöger E. Tervaniemi M. Näätänen R. (1992). Representation of abstract attributes of auditory stimuli in the human brain. Neuroreport 3, 1149–1151. 10.1097/00001756-199212000-00030 1493229

[B44] Schmuckler M. A. (1989). Expectation in music: investigation of melodic and harmonic processes. Music Percept. 7, 109–149 10.2307/40285454

[B45] Seppänen M. Hämäläinen J. Pesonen A.-K. Tervaniemi M. (2012). Music training enhances rapid neural plasticity of n1 and p2 source activation for unattended sounds. Front. Hum. Neurosci. 6:43. 10.3389/fnhum.2012.00043 22435057 PMC3303088

[B46] Tervaniemi M. Huotllainen M. Brattico E. Ilmoniemi R. J. Reinikainen K. Alho K. (2003). Event-related potentials to expectancy violation in musical context. Music. Sci. 7, 241–261 10.1177/102986490300700203

[B47] Tervaniemi M. Kujala A. Alho K. (1999). Functional specialization of the human auditory cortex in processing phonetic and musical sounds: a magnetoencephalographic (MEG) study. Neuroimage 9, 330–336. 10.1006/nimg.1999.0405 10075902

[B48] Tillmann B. Janata P. Bharucha J. J. (2003). Activation of the inferior frontal cortex in musical priming. Brain Res. Cogn. Brain Res. 16, 145–161. 10.1016/s0926-6410(02)00245-8 12668222

[B49] Tremblay K. L. Shahin A. J. Picton T. Ross B. (2009). Auditory training alters the physiological detection of stimulus-specific cues in humans. Clin. Neurophysiol. 120, 128–135. 10.1016/j.clinph.2008.10.005 19028139 PMC2654261

[B50] Vaughan H. G. Ritter W. (1970). The sources of auditory evoked responses recorded from the human scalp. Electroencephalogr. Clin. Neurophysiol. 28, 360–367. 10.1016/0013-4694(70)90228-2 4191187

[B51] Westbury C. F. Zatorre R. J. Evans A. C. (1999). Quantifying variability in the planum temporale: a probability map. Cereb. Cortex 9, 392–405. 10.1093/cercor/9.4.392 10426418

[B52] Wiggs C. L. Martin A. (1998). Properties and mechanisms of perceptual priming. Curr. Opin. Neurobiol. 8, 227–233. 10.1016/s0959-4388(98)80144-x 9635206

[B53] Wise R. J. Scott S. K. Blank S. C. Mummery C. J. Murphy K. Warburton E. A. (2001). Separate neural subsystems within “Wernicke”s area’. Brain 124, 83–95. 10.1093/brain/124.1.83 11133789

[B54] Zatorre R. J. Perry D. W. Beckett C. A. Westbury C. F. Evans A. C. (1998). Functional anatomy of musical processing in listeners with absolute pitch and relative pitch. Proc. Natl. Acad. Sci. U S A 95, 3172–3177. 10.1073/pnas.95.6.3172 9501235 PMC19714

[B55] Zhang F. Eliassen J. Anderson J. Scheifele P. Brown D. (2009). The time course of the amplitude and latency in the auditory late response evoked by repeated tone bursts. J. Am. Acad. Audiol. 20, 239–250. 10.3766/jaaa.20.4.4 19927696

